# Identifying the Predictors of Short Term Weight Loss Failure after Roux-En-Y Gastric Bypass

**DOI:** 10.1155/2022/2685292

**Published:** 2022-10-26

**Authors:** Effat Bahadori, Ali Jafarzadeh Esfehani, Leila Sadat Bahrami, Mohammad Reza Shadmand Foumani Moghadam, Ali Jangjoo, Mohsen Nematy, Afshin Roghani, Reza Rezvani

**Affiliations:** ^1^Department of Nutrition, Faculty of Medicine, Mashhad University of Medical Sciences, Mashhad, Iran; ^2^Metabolic Syndrome Research Center, Mashhad University of Medical Sciences, Mashhad, Iran; ^3^Department of Nutrition Sciences, Varastegan Institute for Medical Sciences, Mashhad, Iran; ^4^Surgical Oncology Research Center, Imam Reza Hospital, Faculty of Medicine, Mashhad University of Medical Sciences, Mashhad, Iran; ^5^Institute for Sustainable Horticulture (ISH), 20901 Langley Bypass, Langley, BC V3A 8G9, Canada

## Abstract

**Introduction:**

Gastric bypass surgery is a gold standard therapy for severe obesity. This study aimed to evaluate anthropometric predictors for short-term excess weight loss (EWL) after Roux-en-Y gastric bypass surgery (RYGB) in a sample of severely obese patients.

**Materials and Methods:**

This cohort study was conducted on severely obese candidates for RYGB bariatric surgery in Mashhad, Iran. Indirect calorimetry, anthropometric measurements, and body composition data were collected before, one, and six months after RYGB.

**Results:**

Fifty-four participants (43, 79.6% women and 11, 20.4% men) with a mean age of 39.63 ± 9.66 years participated in this study. The mean total weight and BMI loss within six months were 32.89 ± 20.22 kg and 12.37 ± 7.34 kg/m^2^, respectively. The mean reduction in adipose tissue and fat-free mass was 24.49 kg and 7.46 kg, respectively. The mean resting metabolism rate (RMR) reduction at one and six months after RYGB was 260.49 kcal and 396.07 kcal, respectively. There was a significant difference in mean RMR between the baseline and one and six months after RYGB (*p* < 0.001). There was no significant gender difference in mean weight and BMI loss percentage at six months post-RYGB (*p* > 0.05). Baseline skeletal muscle mass (SMM), excess BMI loss (EBMIL) at first month after surgery, and baseline neck circumference (NC) could predict EWL six months after surgery.

**Conclusion:**

Reduced RMR shortly after RYGB may be due to FFM reduction. Some anthropometric and their acute changes after RYGB may predict the short-term EWL in RYGB patients.

## 1. Introduction

Obesity is the fifth cause of mortality and morbidity among the most common metabolic disorders in the world [1, 2]. In recent decades, a growing trend in the prevalence of obesity has been observed in developing countries. For instance, the incidence of obesity among Iranian men increased from 10.3% in 2000 to 19.3% in 2016, while the incidence of obesity in Iranian women increased from 19.3% in 2000 to 32.2% in 2016 [3]. Besides, obesity is a predisposing factor for chronic health conditions, including cardiovascular diseases, dyslipidemia, and diabetes [4].

Severe obesity is defined as body mass index (BMI) > 40 kg/m^2^ in individuals with no coseverities or BMI > 35 kg/m^2^ in individuals with coseverities [5]. Severe obesity has had a significant economic and health burden on societies [4, 6]. Bariatric surgery is the most effective way to achieve sustainable long-term weight loss and improve obesity-related health issues [7]. Roux-en-Y gastric bypass (RYGB) and sleeve gastrectomy are the most common surgical treatments for severe obesity [7–9].

Regardless of its effectiveness, bariatric surgery is not always successful in weight reduction and weight maintenance. A common definition for bariatric surgery failure is excess weight loss (EWL) of less than 50% after one year [10]. The failure rate of bariatric surgery is reported to be as high as 20% [11]. However, variations in the definition of failure have made it difficult to reach a consensus regarding bariatric surgery failure and its predicting factors. Bariatric surgery failure might be due to technical complications, personal factors, predisposing and underlying conditions, and appetite changes [12]. Therefore, patient selection is of great importance in treatment success using bariatric surgery [12]. Although various predicting factors have been reported for success in achieving 50% EWL one year after surgery, there is no common opinion about using these factors in the patient selection [10]. Some of these predictors include younger age, lower BMI before surgery, and lack of mental disorders, including stress and anxiety [13–17]. Considering the differences in the surgical techniques and facilities, it can be hypothesized that treatment failure might defer between different hospital settings and countries. Therefore, evaluating the predictors for bariatric surgery success should be conducted regionally. As chronic patients including patients with severe obesity are referred to surgeons through the Primary Health Care (PHC) system in Iran, identifying easy and accessible predictors for bariatric surgery failure is important. Therefore, this study aimed to evaluate anthropometric predictors for short-term EWL after RYGB in a sample of severely obese patients in Mashhad, Iran.

## 2. Methods

### 2.1. Study Population

Participants were randomly selected from those who were a candidate for RYGB surgery in a bariatric surgery clinic in Mashhad, Iran. Fifty-four patients, including 43 women (79.6%) and 11 men (20.4%) aged 18–65 years old, were recruited. Inclusion criteria were BMI ≥ 40 kg/m^2^, or BMI ≥ 35 kg/m^2^ with two or more comorbidities, including metabolic syndrome based on the Adult Treatment Panel III (ATP III) criteria, type 2 diabetes, insulin resistance, acanthosis nigricans, fatty liver disease, sleep apnea, polycystic ovary syndrome, peripheral vascular disease, and progressive varicose. Subjects were excluded if they were performing a professional exercise, were on a special diet, drug addict, alcohol abusers, or smokers. Written informed consent was obtained from all the participants before the beginning of the study. All patients were informed about the advantages and disadvantages of RYGB as well as postoperative medical and nutritional care.

### 2.2. Sample Size

The sample size was calculated based on the findings of indirect calorimetry studies after bypass surgery using the following equation [18]. In this equation, *Z*_1 − *α*/2_ is the critical value of the normal distribution at 1 − *α*/2, *Z*_1 − *β*_ is the critical value of the normal distribution at 1 − *β*, *σ*_1_ and *σ*_2_ are the population variance, and *µ*_1_ and *µ*_2_ are the mean of the outcome value in groups.

(1) $$\begin{array}{c} n=\frac{{\left(z_{1}-\left(\alpha /2\right)+z_{1}-\beta \right)}^{2}\left(\sigma_{1}^{2}+\sigma_{2}^{2}\right)}{{\left(\mu_{1}-\mu_{2}\right)}^{2}}\text{.} \end{array}$$

The calculated sample size was 53 patients which was increased to 60 considering 10% drop-out.

### 2.3. Anthropometric and Body Composition Assessment

Anthropometric data, including height (cm), weight (kg), waist circumference (cm), hip circumference (cm), and body composition data, were collected at the baseline (before RYGB) and the 1^st^ and 6^th^ months after RYGB. Height was measured to the nearest 0.1 cm in the standing position with bare feet and head in the Frankfurt position. Body weight was measured in light clothing without shoes by using a calibrated digital scale. Waist circumference (WC) was measured horizontally at midway between the lowest rib and the iliac crest with nonelastic meters. Hip circumference (HC) was measured horizontally at the widest diameter of the buttocks. Waist-hip ratio (WHR) was calculated by dividing WC to hip circumference (HC). Body mass index (BMI, kg/m^2^) was calculated by dividing weight (kg) by the square of height (m). Body water (L), skeletal muscle mass (kg), body cell mass (kg), bone minerals, soft body tissue (kg), fat mass (kg), fat percentage, abdominal fat percentage, and FFM (kg) were determined based on bioelectric impedance analysis using Inbody 770 BIA unit (Inbody Co., Ltd., Seoul, South-Korea). For a more accurate assessment of body composition, subjects were asked to drink enough water (4–6 glasses), abstain from all foods and beverages for 2 to 3 hours, and avoid exercise 4 to 6 hours before the measurement [19].

### 2.4. Blood Pressure Measurement

Changes in the systolic and diastolic blood pressure were measured for all subjects at the baseline, 1^st^, and 6^th^ month follow-up using a sphygmomanometer with a proper cuff size. Measurements were performed while seated after 20 minutes of rest.

#### 2.4.1. Dietary Intake Assessment

The dietary intake was assessed before surgery based on a three-day dietary record. Dietary intakes of macronutrients and micronutrients were analyzed using Nutritionist 4 software (First Databank Inc., Hearst Corp., San Bruno, CA).

#### 2.4.2. Resting Metabolic Rate Assessment

RMR was determined by indirect calorimetry (Cortex Metalyzer 3B, Germany). Measurement was performed after at least 20–30 minutes of rest. For determining a more precise RMR, subjects were asked to stay hydrated, fast for 4 to 12 hours, avoid tobacco and narcotic drugs, caffeine, and carbonated beverages, and perform vigorous activities for 4 hours before performing the test. The calculated RMR was adjusted for body weight (kg) by dividing RMR by weight (RMR/kg). RMR was calculated at the baseline and 1^st^ and 6^th^ month follow-up.

### 2.5. Weight Loss Assessment after RYGB Surgery

Percentage of total weight loss (%TWL), excess BMI loss percentage (%EBMIL), and excess weight loss percentage (%EWL) were calculated based on the amount of weight loss one and six months after surgery using the following equations [20]. The ideal BMI was considered BMI = 25 kg/m^2^:

  %TWL = [(baseline weight − weight six months after surgery) ÷ (baseline weight)] × 100
  %EBMIL = [(the amount of BMI reduction six months after surgery) ÷ (baseline BMI − ideal BMI)] × 100
  %EWL = [(the amount of weight loss six months after surgery) ÷ (baseline weight − ideal weight)] × 100.

### 2.6. Statistical Analysis

We used the statistical package for social sciences (SPSS) software version 16 to analyze the data. The normality of data was checked using the Kolmogorov–Smirnov and Shapiro–Wilk tests. Mean ± standard deviation (SD) was reported for normally distributed data, and median (interquartile range) was reported for non-normally distributed data. The independent *t*-test and Mann–Whitney tests were used to compare normally distributed and non-normally distributed continuous variables between the groups, respectively. Data analysis was performed based on the intention to treat analysis. The missing values were imputed using multiple imputations based on age, gender, BMI, FM, and FFM. The best imputation model for the missing variables was chosen. Comparison of the continuous variables between study periods was performed using repeated-measures analysis of variance (ANOVA) with Bonferroni correction. Regression trees were drawn using the rpart package in R version 4.3.1 (R Core Team, 2020) to visualize the predictive models for EWL% [21]. The regression trees were used to split the data based on cut-offs for key variables (decision points) to differentiate individuals in terms of successful EWL. Various regression trees were developed and were used to form regression trees. The regression trees yielded relatively consistent results. The results of the regression tree were used to identify the features (a synonym for variables in statistics) that are most related to the EWL. The identified features based on the decision tree findings were used to perform a regression model to predict the EWL% based on the baseline and one-month variables. The *p* and beta values as well as 95% confidence interval for beta were reported for the regression model. The beta value indicates the amount of change in the dependent variable (EBWL%) for each one unit increase in the value of the independent variable. The level of statistical significance was considered *p* < 0.05.

## 3. Results

A total of 62 participants were identified among whom, 6 did not consent to participate and the data for 2 participants were missing. Therefore, 54 participants (11, 20.4% males and 43, 79.6% females) entered the study. All participants were followed up throughout the study duration. The mean age of the participants was 39.63 ± 9.66 years. Baseline characteristics of the study participants are presented in Table 1. There was no significant difference in mean age and BMI between genders at the baseline, while the height and weight of men were significantly higher than women (*P* < 0.001 each) (Table 1).

Comparison of anthropometric and body composition variables between the baseline and the 1^st^ and 6^th^-month follow-up are presented in Table 2. All anthropometric and body composition variables significantly change at the 1^st^ and 6^th^-month follow-up compared to the baseline except for WHR at the 1^st^-month follow-up (*p*=0.471).

A comparison of crude RMR and RMR adjusted for body weight and FFM between the baseline and the 1^st^ and 6^th^-month follow-up is presented in Table 3. There was a significant difference in crude RMR and RMR/weight between the baseline and the 1^st^ and 6^th^ month follow-up (Table 3).

**Figure 1:**
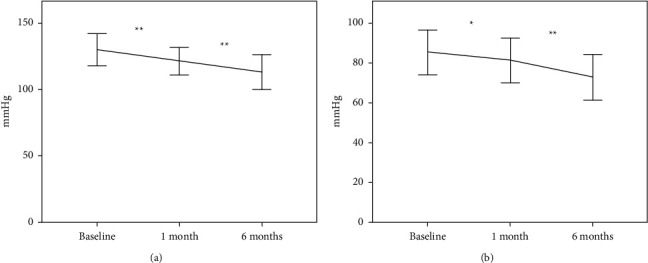
Changes in systolic (a) and diastolic (b) blood pressure during the study period. Fifty-four participants were evaluated in all the three time points. ^∗^*P*=0.020, ^∗∗^*p* < 0.001.

The changes in systolic and diastolic blood pressure during the study period are presented in Figure 1. There was a significant difference in systolic and diastolic blood pressure between the baseline and the 1^st^ (*p* < 0.001 and *p*=0.020, respectively) and 6^th^ month (*p* < 0.001 each) follow-up (Figure 1). The WL%, EBMIL%, and EWL% from the baseline to the 1^st^ month follow-up and fromthe baseline to the 6^th^ month follow-up are shown in Table 4.

**Figure 2:**
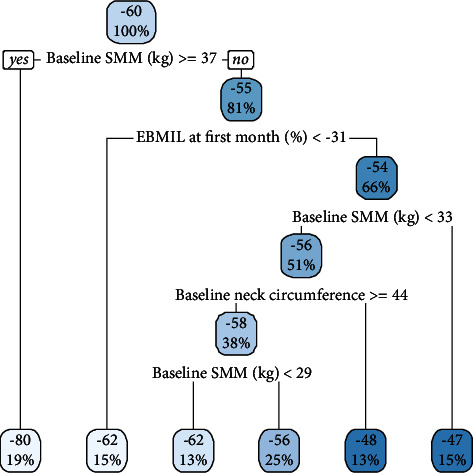
Decision tree for predicting weight loss failure during the first 6 months after surgery. The values in the top of the squares indicate excess weight loss percentage, and the values in the bottom of the squares indicate the percentage of the participants with the outcome. The right branches should be followed if the condition is met, while the left branches should be followed if the condition is not met.

The regression tree (Figure 2) was drawn to identify the predictors of failure to lose weight after 6 months. Based on the regression tree, participants with SMM of at least 37 kg lost 80% of the EWL at the 6^th^-month follow-up, while participants with baseline SMM between 29 and 33 kg were at high risk of weight loss failure after 6 months. Fast reduction in EBMIL was related to weight loss failure in participants with neck circumference lower than 44 cm, while participants with higher neck circumference and lower baseline SMM were more likely to lose more than 50% of the excess weight within 6 months.

Based on the regression tree results, a regression model was created to identify the predictors of EWL%. The regression analysis showed that baseline SMM was the only predictor for EWL% in the 6^th^ month (Table 5). This finding indicated that for each one kg increase in baseline SMM, EWL% is reduced by 1.418 percent (negative beta value indicates negative relationship).

## 4. Discussion

This study included 54 subjects with severe obesity which had undergone RYGB surgery. RYGB resulted in average weight, EWL, and EBMIL of 16.71, 49.05, and 58.72%, respectively. Losing 30% of their initial weight, 28% of their body mass index, and 16 cm of their waist circumference after 6 months of gastric bypass surgery, the findings of this study also showed that although the crude BMR significantly reduced during the study period, the weight-adjusted BMR (BMR/weight) significantly increased over time. RYGB also reduced all anthropometric measurements except for WHR during the 6 months of follow-up. The findings of this study highlighted the role of skeletal muscle mass in short-term weight reduction after RYGB, which was more prominent compared to EBMIL.

The main aim of weight loss interventions is fat tissue reduction while maintaining lean mass to improve the nutritional and metabolic status and maintain physical strength [22]. However, lean body mass loss is inevitable, especially in the first 3 to 6 months after operation [23]. Ciangura et al. [22] examined 42 women with severe obesity and found that 30% of the initial weight loss during the six months after surgery was related to a reduction in body fat-free tissue [22]. The findings of our study also indicated a significant reduction in SMM and FFM, but the extent of the lean body mass loss was less than fat mass reduction. This finding may indicate good postoperative nutrition management.

In addition, the highest rate of weight loss and loss of lean body mass was observed during the first month after surgery, and fat-free mass appeared to be somewhat preserved after this time. It was previously shown that nearly one-third (31%) of the weight loss in the first year after gastric bypass surgery could be attributed to lean body tissue [24]. Previous studies indicated a higher rate of lean tissue loss in the first four to six weeks after gastric bypass surgery compared to fat mass [25–28]. These findings were in line with the results of our study.

The results of this research showed that most changes in body composition occurred in the first month after gastric bypass surgery. Changes in body composition can be interpreted as postoperative adaptation. Energy adaptations in response to RYGB bariatric surgery in people with severe obesity were associated with changes in insulin, leptin, adiponectin, T3, intestinal hormones, and SNS activity [29]. The energy compatibility in TEE and its components resulting from RYGB surgery were observed after surgery. The results of some studies showed that most thermogenesis adaptations occur one and a half months (45 days) after surgery, which coincides with a decrease in FFM and biochemical changes. It was indicated that muscle wasting increased after bariatric surgery when categorizing participants based on sarcopenia. Considering the definition used for sarcopenia based on the skeletal muscle index, all the participants in the current study had sarcopenic obesity [30]. It was previously reported that body composition especially muscle mass was related to bariatric surgery success [26].

Our study showed that crude RMR decreased 6 months after surgery compared to the baseline, which may support the hypothesis that the reduction in RMR short term after RYGB surgery was due to metabolic adaptation in response to FFM reduction [31, 32]. However, BMR is considered an inaccurate marker in evaluating weight change and success after bariatric surgery. As the extent of fat mass and weight reduction is higher than the extent of BMR reduction, the weight-adjusted BMR tends to increase after surgery [33–36]. It was also shown that changes in BMR are associated with weight regain after bariatric surgery [37, 38]. However, the regression tree in our study failed to identify regression cut-off for neither BMR nor BMR/kg in determining EWL during the first 6 months after RYGB. This finding might be related to the small sample size and the presence of other confounders including the physical activity level, medication use, and the high prevalence of sarcopenia among the study participants. On the other hand, due to the observed low muscle mass in the participants of the current study, it is rational that the changes in RMR or RMR/kg were related to other factors, whose effects on BMR were not as strong as muscle mass and thus could not reach a statistical significance.

Based on the regression tree findings, an increase in preoperative SMM was associated with increased EWL, while in participants with lower SMM, EWL was related to the extent of obesity (neck circumference) and pace of weight reduction (EBMIL in the first month after surgery). Fast weight reduction might be associated with increased muscle mass reduction, which in part can reduce BMR. It was previously shown that NC was a predictor of severe sleep apnea [39, 40]. A study suggested 34.5 cm as the cut-off value for NC in predicting obstructive sleep apnea [41]. Improving sleep apnea was shown to reduce BMR in both animal and human studies [42, 43]. However, a systematic review showed that improving obstructive sleep apnea with continuous positive airway pressure reduced leptin and ghrelin levels which could reduce food intake [43]. Considering the mean preoperative NC in the current study (47.91 cm), it can be deduced that the study participants had degrees of obstructive sleep apnea at the baseline that might have improved during the study duration. Taking into account the baseline SMM, the combination of very low preoperative SMM and high NC (obstructive sleep apnea) may have resulted in acceptable EWL (−62% to −56%) due to the extent of FM reduction. On the contrary, low preoperative SMM along with lower NC was associated with unfavorable EWL at 6 months. This finding might indicate that the effect of sleep apnea on BMR can differ based on muscle mass; however, the classification cut-off for preoperative NC was above the cut-off for predicting obstructive sleep apnea, which might be the reason for the observed nonsignificant relationship between NC and EWL in the regression analysis.

There are some limitations in our study. This study was designed as a preliminary study to evaluate RMR and body composition in obese patients undergoing RYGB surgery. Therefore, the sample size might not be large enough to generalize the results to the whole population. It is suggested that these variables be assessed in larger studies. Furthermore, although the SMM and FFM could be considered indirect measures of the level of physical activity, the current study did not take into account the level of physical activity of study participants. It is recommended that further studies evaluate the effect of RYGB on different levels of sarcopenia and muscle mass.

## 5. Conclusion

Based on the findings of the current study, it seems that in the short term, RYGB has the most significant impact on weight reduction in individuals with higher preoperative muscle mass and that fast reduction in excess BMI was related to unfavorable outcomes 6 months after RYGB. Neck circumference was also a predictor for favorable excess weight lossafter RYGB.

## Figures and Tables

**Table 1:** Baseline characteristics of the study participants categorized by gender.

Variables	Total population (*n* = 54)	Male (*n* = 11)	Female (*n* = 43)	*P* value
Age (years)	39.63 ± 9.66	34.82 ± 7.98	40.86 ± 9.75	0.06
Height (cm)	162.02 (8.18)	177.25 ± 8.33	160.68 ± 4.92	<0.001^∗^
Weight (kg)	121.81 ± 17.64	138.44 ± 17.88	118.55 ± 15.01	<0.001^∗^
BMI (kg/m^2^)	45.44 ± 21.26	45.20 ± 4.71	45.50 ± 6.58	0.88

Abbreviations: BMI: body mass index. The data are presented as the mean ± STD, and the independent sample *t*-test was used for the comparison. ^∗^indicates significant difference between genders.

**Table 2:** Comparison of anthropometric measurements at the baseline, one month, and six months after gastric bypass surgery among study participants.

Variables	Baseline (*n* = 54) mean ± SD	1^st^ month (*n* = 54) mean ± SD	6^th^ month (*n* = 54) mean ± SD	*p*	Baseline vs. 1^st^ month		Baseline vs. 6^th^ month		1^st^ month vs. 6^th^ month	
					Mean difference (SE)	*p*	Mean difference (SE)	*p*	Mean difference (SE)	*p*
Weight (kg)	122.60 ± 17.22	107.65 ± 12.97	89.15 ± 9.53	<0.001^∗^	−14.95 (1.35)	<0.001^∗^	−33.45 (1.77)	<0.001^∗^	−18.49 (1.35)	<0.001^∗^

BMI (kg/m^2^)	45.47 ± 6.19	40.57 ± 5.08	33.61 ± 3.39	<0.001^∗^	−4.91 (0.57)	<0.001^∗^	−11.86 (0.64)	<0.001^∗^	−6.96 (0.52)	<0.001^∗^

FM (kg)	62.51 ± 10.71	53.72 ± 9.98	38.34 ± 7.30	<0.001^∗^	−8.79 (0.85)	<0.001^∗^	−24.17 (1.19)	<0.001^∗^	−15.37 (1.06)	<0.001^∗^

FM (%)	51.23 ± 4.76	49.60 ± 6.12	43.59 ± 6.14	<0.001^∗^	−1.63 (0.42)	<0.001^∗^	−7.64 (0.77)	<0.001^∗^	−6.01 (0.93)	<0.001^∗^

FFM (kg)	59.43 ± 10.84	53.76 ± 8.75	50.43 ± 7.54	<0.001^∗^	−5.67 (0.78)	<0.001^∗^	−9.01 (1.12)	<0.001^∗^	−3.33 (1.09)	0.003^∗^

SMM (kg)	32.88 ± 6.58	29.62 ± 5.19	27.56 ± 4.53	<0.001^∗^	−3.26 (0.52)	<0.001^∗^	−5.32 (0.68)	<0.001^∗^	−2.07 (0.67)	0.003^∗^

WHR	0.98 ± 0.13	1.00 ± 0.12	0.96 ± 0.13	0.160	0.02 (0.02)	0.999	−0.02 (0.02)	0.939	−0.04 (0.02)	0.135

NC (cm)	47.91 ± 5.75	44.28 ± 3.71	38.13 ± 3.54	<0.001^∗^	−3.64 (0.53)	<0.001^∗^	−9.78 (0.88)	<0.001^∗^	−6.14 (0.64)	<0.001^∗^

Chest C (cm)	121.31 ± 8.78	114.48 ± 6.58	103.20 ± 5.99	<0.001^∗^	−6.84 (0.75)	<0.001^∗^	−18.11 (1.19)	<0.001^∗^	−11.27 (1.06)	<0.001^∗^

Ab C (cm)	123.91 ± 15.78	120.11 ± 9.96	104.15 ± 9.50	<0.001^∗^	−3.80(1.51)	0.015^∗^	−19.76(1.71)	<0.001^∗^	−15.96 (1.01)	<0.001^∗^

HC (cm)	126.50 ± 9.33	118.41 ± 6.55	110.01 ± 5.18	<0.001^∗^	−8.09 (0.74)	<0.001^∗^	−16.49(0.96)	<0.001^∗^	−8.40 (0.64)	<0.001^∗^

Abbreviations: BMI: body mass index; FM: fat mass; FFM: fat-free mass; SMM: skeletal muscle mass; WHR: waist-to-hip ratio; NC: neck circumference; Chest C: chest circumference; Ab C: abdominal circumference; HC: hip circumference. Data were presented as the mean ± standard deviation (SD) or mean difference and standard error (SE). The repeated-measures analysis of variance was performed for the comparison. Group comparison was performed using Bonferroni correction. ^∗^Significant difference.

**Table 3:** Comparison of the resting metabolic rate, metabolic and calorimetric factors at the baseline, 1 month, and 6 months after gastric bypass surgery among study participants.

	Baseline (*n* = 54) mean ± SD	1^st^ month (*n* = 54) mean ± SD	6^th^ month (*n* = 54) mean ± SD	Time effect *p*	Baseline vs. 1^st^ month		Baseline vs. 6^th^ month		1^st^ month vs. 6^th^ month	
					Mean difference (SE)	*p*	Mean difference (SE)	*p*	Mean difference (SE)	*p*
RMR (kcal)	1669 ± 250.16	1533.06 ± 188.19	1460.09 ± 160.03	<0.001^∗^	−136.12 (20.77)	<0.001^∗^	−209.09 (27.90)	<0.001^∗^	−72.97 (22.65)	0.002^∗^

RMR/weight (kcal/kg)	13.68 ± 1.41	14.32 ± 1.44	16.44 ± 1.44	<0.001^∗^	0.64 (0.16)	<0.001^∗^	2.76 (0.20)	<0.001^∗^	2.13 (0.19)	<0.001^∗^

Abbreviations: RMR: resting metabolic rate; SD: standard deviation. Data were presented as mean ± SD. ^∗^Significant difference.

**Table 4:** Comparison of WL%, EBMIL%, and EWL% between the baseline and the 1^st^ month follow- up and the baseline and the 6^th^ month follow-up among study participants.

Variables	1^st^ month (*n* = 54)	6^th^ month (*n* = 54)	*p*
WL%	−10.66 (5.22)	−16.71 + 8.15	<0.001^∗^
EBMIL%	−22.33 (12.64)	−58.72 (17.23)	<0.001^∗^
EWL%	19.46 (11.31)	49.05 + 17.62	<0.001^∗^

Abbreviations: TWL%: percentage of total weight loss, EBMIL%: percentage of excess body mass index loss, EWL%: percentage of excess weight loss. The data are presented as the mean ± STD. The Wilcoxon test was used for the comparison. ^∗^Significant difference.

**Table 5:** Relationship between EWL% and baseline skeletal muscle mass and excess body mass index loss after one month in study participants.

Variables	*p*	*β*	95% CI for *β*	
			Lower	Upper
Baseline SMM	<0.001^∗^	−1.418	−2.009	−0.827
EBMIL in 1^st^ month	0.730	−0.027	−0.180	0.127
Baseline neck circumference	0.181	0.433	−0.209	1.075

Abbreviations: SMM: skeletal muscle mass, EBMIL: excess body mass index loss, CI: confidence interval.

## Data Availability

Authors can also make data available on request through a data access committee, institutional review board, or the authors themselves.

## Abbreviations

Ab C: Abdominal circumference
ANOVA: Analysis of variance
BMI: Body mass index
BMR: Basal metabolic rate
Chest C: Chest circumference
CI: Confidence interval
Cm: Centimeter
EBMIL: Excess body mass index loss
EWL: Excess weight loss
FFM: Fat free mass
FM: Fat mass
HC: Hip circumference
Kg: Kilogram
NC: Neck circumference
RMR: Resting metabolic rate
RYGB: Roux-en-Y gastric bypass surgery
SD: Standard deviation
SE: Standard error
SMM: Skeletal muscle mass
TEE: Total energy expenditure
TWL: Total weight loss
WC: Waist circumference
WHR: Waist-to-hip ratio.
